# Treating recurrent hemarthrosis after knee arthroplasty with selective embolization: a cohort study of 56 patients

**DOI:** 10.2340/17453674.2024.42660

**Published:** 2025-01-09

**Authors:** Suvi-Maria SIROLA, Juuso HEIKKINEN, Pekka KERIMAA, Juho KARINIEMI, Tuukka NIINIMÄKI

**Affiliations:** 1 University of Oulu, Faculty of Medicine, Oulu; 2 Oulu University Hospital, Department of Orthopedics and Traumatology, Oulu; 3 Oulu University Hospital, Department of Radiology, Oulu, Finland

## Abstract

**Background and purpose:**

Recurrent hemarthrosis (RH) is a rare late complication of knee arthroplasty, with an unknown etiology. We aimed to evaluate the effectiveness of arterial embolization (AE) on resolution of hemarthrosis following knee arthroplasty. Additionally, we investigated pain management requirements after the procedure and related complications.

**Methods:**

56 patients underwent AE for recurrent hemarthrosis between 2015 and 2023. The prevalence of hemarthrosis was 0.6%. The median age of the patients was 70 years (range 42–88), with 41 females and 15 males. 70 embolizations were performed, consisting of 56 initial procedures and 14 repeat procedures. Clinical success was defined as the resolution of hemarthroses.

**Results:**

Technical success was achieved in 93% of cases. Clinical success improved from 64% to 79% after the second treatment; subsequent sessions did not yield further improvement. 12 patients (21%) required 1 or more reoperations. The majority of patients (86%) relied solely on analgesics for post-treatment pain management. Complications occurred in 7% of treatments, most of which resolved spontaneously.

**Conclusion:**

AE is effective in the treatment of recurrent hemarthrosis but 21% had reoccurance. Oral analgesics are generally sufficient for managing post-embolization pain. 7% had complications.

The most common complications of knee arthroplasty are infection, deep vein thromboembolism, periprosthetic fracture, prosthetic loosening, and hemarthrosis [1]. Recurrent hemarthrosis is a rare, late, and disabling complication following knee arthroplasty, with a reported prevalence of up to 1.6% [2-4]. It involves bleeding of the vascular synovia, leading to knee joint effusion and pain; however, the etiology of recurrent hemarthrosis remains unclear in most patients [5,6]. While nonoperative and surgical treatments are available, nonoperative approaches show effectiveness in only a minority of cases [2,3,5]. Selective endovascular embolization of arteries has been proposed as an alternative to traditional surgical methods [5,7]. This minimally invasive technique involves injecting embolic agents through a catheter into the relevant arteries to reduce blood flow to hypertrophic synovial tissue [5].

Due to the rarity of recurrent hemarthrosis, the number of research articles on the subject is relatively low, primarily comprising case reports with limited sample sizes, typically involving fewer than 10 patients [8]. The efficacy and modality of selective embolization have been predominantly highlighted in these case reports.

We aimed to evaluate the effectiveness of arterial embolization (AE) on resolution of hemarthrosis following knee arthroplasty. Additionally, we investigated pain management requirements after the procedure and related complications.

## Methods

### Study design

This is a retrospective study of all patients who underwent AE at the Oulu University Hospital from 2015 to 2023. Patients were identified using the NeaRIS radiology management system. Admissive units were defined as orthopedic and rheumatology wards and outpatient clinics. Patients with RH after KA were identified by cross-referencing the outpatient clinic register with the ICD-10 code M25.0 (hemarthrosis). Inclusion criteria comprised a history of KA (NGB10–60) and recurrent hemarthrosis [9]. Recurrent hemarthrosis was defined as 2 or more bleeding episodes characterized by recurrent acute swelling, and bleeding was confirmed by joint aspiration. Exclusion criteria involved traumatic and immediate postoperative causes of hemarthrosis (< 4 weeks postoperatively) and obvious mechanical reasons for hemarthrosis needing revision surgery. Our study complies with and is reported according to STROBE guidelines [10].

### Patients

Patient data were reviewed retrospectively and information on patient age, sex, history of previous arthroplasties, potential rheumatoid arthritis diagnosis, blood thinner use, and the timing of symptoms and treatment initiation were recorded. Subsequently, we compiled data on the implant, operation date, diagnosis, and the type of the operation (primary or reoperation).

The collected variables of the embolization procedures were the date(s), number of embolizations, types of embolization materials used, and possible reoperations after the last embolization. Subsequently, we evaluated the possible complications of embolization as none, mild, moderate, or severe [11]. Mild complications resolved spontaneously, whereas moderate complications necessitated elective treatment or procedures. Serious complications would require immediate intervention. Finally, we examined the overall duration of hospital stay and pain management, both before and after embolization.

### Practice of AE

When hemarthrosis first occurred the knee was aspirated, but after recurrence the patient was referred by a general practitioner to an orthopedic surgeon for further evaluation. At our hospital, selective embolization was chosen as the first-line treatment for refractory cases. If symptoms recurred, embolization could be repeated. If recovery was not achieved with embolizations, reoperation was indicated.

**Figure 1:**
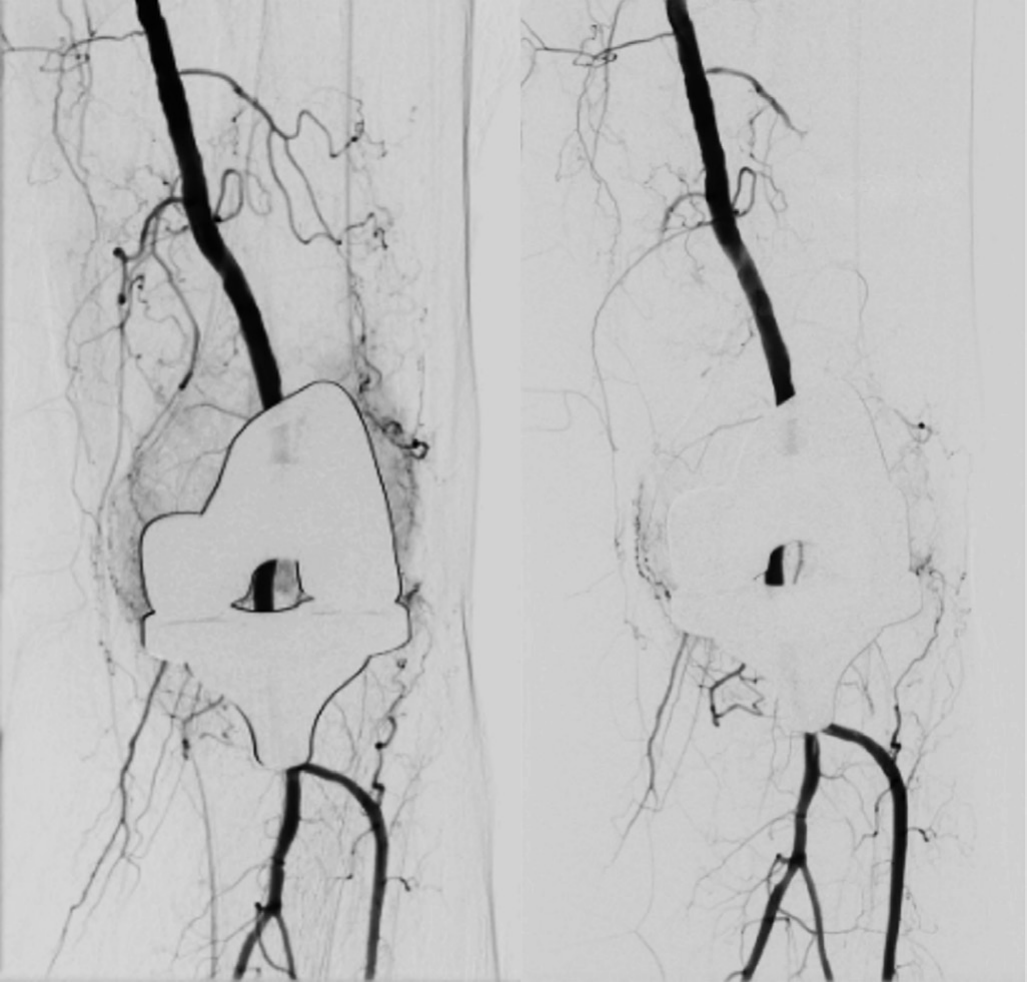
Hypervascular synovium both laterally and medially at baseline. After embolization, no pathological hyperemia was observed.

Embolization procedures were routinely performed under local anesthesia. Midazolam was administered perioperatively upon patient request. All blood thinners, except acetylsalicylic acid, were discontinued. Nonselective angiography of the femoral and popliteal arteries was performed through femoral access to identify areas of hypervascular synovium indicated by pathological vascular hyperemia. Arteries with hyperemia were selected using a microcatheter. Embolization involved the use of tris-acryl gelatin microspheres, polyvinyl alcohol (PVA) particles, liquid embolic agents, and/or coils, chosen at the discretion of the performing interventional radiologist. The embolic agents applied in this study are commonly used and within an empirically validated size range. PVA particles, specifically Contour (Boston Scientific, Marlborough, MA, USA), were the predominant embolization agents (Table 1). The particle sizes ranged from 150 to 355 μm, with 150–250 μm being the most frequently used. In 6 embolizations, various combinations of particles, coils, and tissue glue were utilized. Embolization was performed until the control angiography showed decreased visualization of the hypertrophic synovium (Figure 1).

**Table 1:** Embolization demographics (N = 70)

Factor	Frequency
Embolization agent	
Combination	6
Onyx	2
PVA particles	45
Spherical	17
Analgesia	
Femoral nerve block	10
Peroral analgesia	60
Complications	
None	65
Mild	3
Moderate	2
Severe	0
Hospital stay, days	
0	2
1	56
2	8
3	3
4	1

PVA = polyvinyl alcohol

### Outcomes

Technical success was defined by an interventional radiologist as the elimination or significant reduction of atypical hyperemic blush. Partial success was defined as incomplete or uncertain elimination of hyperemia. Clinical success was defined as the absence of clinical evidence of further hemarthrosis. Clinical success was assessed by the operating surgeon during follow-up. Follow-up was conducted individually via telephone or in-person appointments. Patients without contact via telephone or in-person appointments were retrospectively reviewed from national patient records. All the patients were instructed to contact the hospital if symptoms recurred.

### Statistics

Data is presented as medians and ranges in parentheses.

### Data sharing plan, funding and disclosurest

As this was a retrospective registry study, the requirements for ethical approval and informed consent were waived, and the participants were not contacted. This research was funded by the Oulu University Hospital State Research Fund. This research received no specific grants from any funding agency in the commercial or not-for-profit sector. The legal basis for processing personal data is for public interest and scientific research. The authors declare that they have no conflicts of interest. Complete disclosure of interest forms according to ICMJE are available on the article page, doi: 10.2340/17453674.2024.42660

## Results

**Figure 2:**
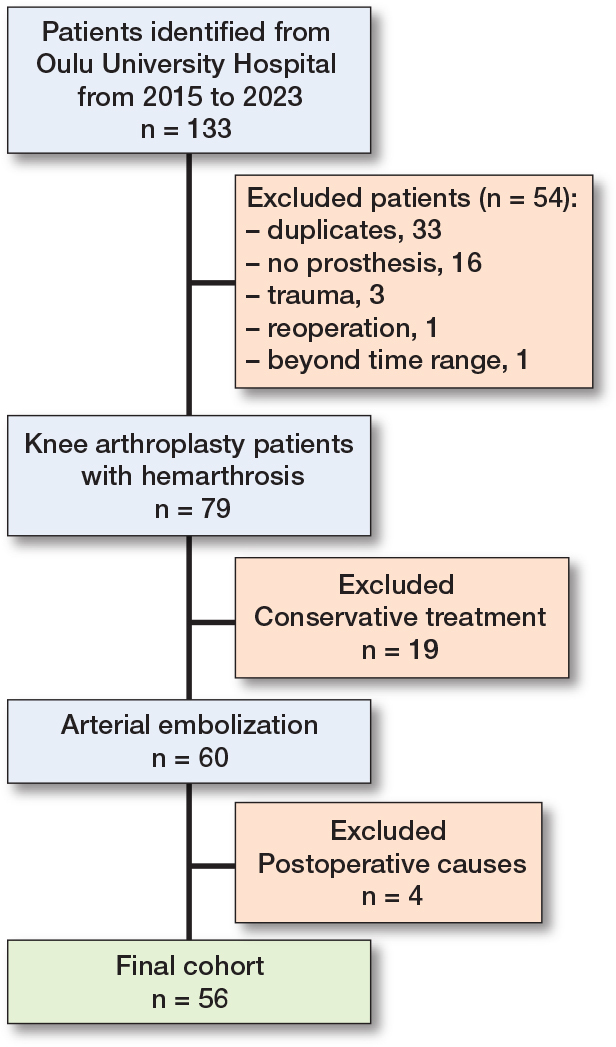
Flowchart of patients. Postoperative causes of hemarthrosis were diagnosed at < 4 weeks postoperatively, including AV fistulae, pseudoaneurysm, and postoperative bleeding.

56 patients were eligible for this study (Figure 2). The patients were predominantly females (73%), and the median age at the time of embolization was 70 years (range 42–88). The majority (96%) had a history of total knee arthroplasty (TKA), while 2 patients had undergone unicompartmental knee arthroplasty (UKA) and 50 (89%) were primary operations. 44 patients had undergone KA at Oulu University Hospital while 12 patients underwent KA in other Finnish hospitals. From 2015 to 2023, 6,867 KAs were identified at the Oulu University Hospital. Based on the number of KAs performed at Oulu University Hospital, the prevalence of hemarthrosis was 0.6%. Primary osteoarthritis (OA) was diagnosed in 54 (96%) patients prior to KA. Only 2 patients had a history of rheumatoid arthritis. Hemarthrosis was diagnosed at a median of 20 (range 1–172) months after KA. At the time of hemarthrosis, 21 patients (38%) had been taking blood-thinning medication.

**Figure 3:**
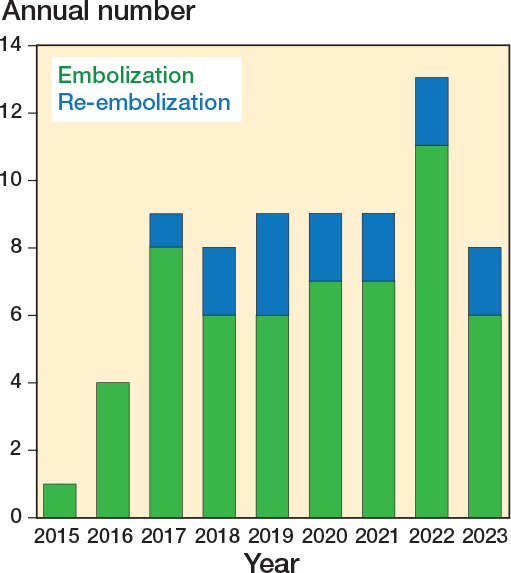
Annual number of embolization procedures: initial vs reinterventions at Oulu University Hospital.

Of the 56 patients, 43 (77%) underwent 1 embolization, 12 (21%) underwent 2 treatments, and 1 (2%) received 4 treatments. The median interval between the first and second embolizations was 82 (range 9–1,224) days. The total number of embolizations has shown an increasing trend since the initiation of the procedure in 2015 (Figure 3).

### Embolization results

**Figure 4:**
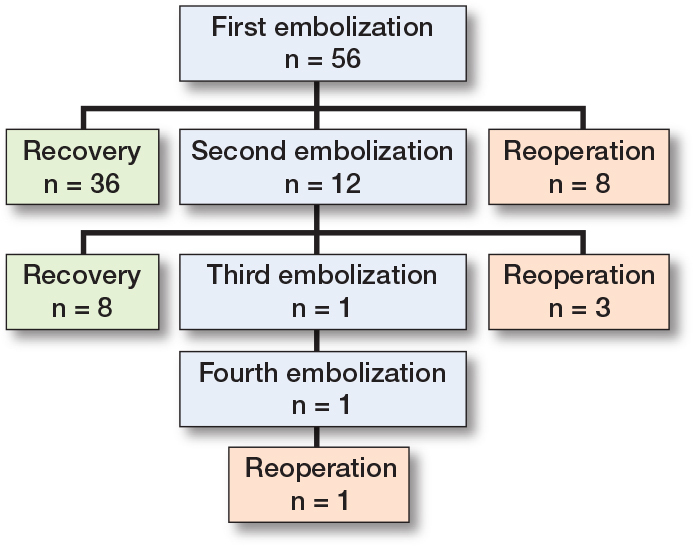
Flowchart of patient outcome.

Technical success was achieved in 65 (93%) cases, with partial success noted in instances involving an area obscured by an endoprosthesis, inadequate patient cooperation, or excessively steep vascular anatomy. Final clinical success was achieved in 44 (79%) patients, with rates of 64% and 79% after the first and second treatments, respectively (Figure 4). No further improvement in clinical success was observed after the second treatment.

### Pain management

Perioperative and postoperative pain management were categorized into 2 groups: analgesics and prophylactic regional anesthesia. Prophylactic femoral nerve blocks were occasionally employed (10 patients). Following embolization, the majority of patients were managed with oral analgesics such as paracetamol or opioids (Table 1). 2 patients (3%) were discharged on the same day. 56 patients (80%) were discharged on the first postoperative day, 8 (11%) on the second postoperative day, 3 (4%) on the third postoperative day, and 1 (1%) on the fourth postoperative day.

### Patient outcome

12 (21%) patients reported recurrent hemarthrotic symptoms after embolization, necessitating reoperation (Figure 4). Only 4 revisions with implants were needed, as smaller operations were more prevalent (Table 2). All reoperations were successful in terms of clinical success, as no recurrence of hemarthrosis was reported.

**Table 2:** Frequencies of reoperations

Procedure	Frequency
Exchange of a modular liner	2
Total revision of knee arthroplasty, cruciate-retaining prosthesis	1
Total revision of knee arthroplasty, semi-constrained or hinge	3
Patellar resurfacing	3
Total synovectomy of knee, arthroscopic	3

### Complications

Complications occurred in 5 (7%) of the treatments (3 mild and 2 moderate). Mild complications included rash, puncture site hematoma, or small fatty necrosis. All mild complications resolved spontaneously without sequelae. Moderate complications involved a puncture site-related pseudoaneurysm of the femoral artery and a necrotic wound. Both moderate complications were treated successfully.

## Discussion

To date, our study is the second-largest case series on this topic. Owing to the rarity of this complication, trials are unlikely to be performed, and knowledge of this treatment method depends on the clinical series presented here.

We aimed to assess the results of selective AE and to evaluate pain following AE for recurrent hemarthrosis. The rate of clinical success per treatment increased from 64% to 79% between the first and second embolization. Complications occurred in 7% of the treatments.

A novel discovery in our study was the notable frequency of repeated embolization and reoperation. Of the 56 patients, 12 required repeat embolizations. Only a few studies have reported a greater rate of repeated embolizations [3,12,13]. However, as highlighted by Van Baardevijk et al. [3] it is plausible that the incidence rate has been under-reported in smaller case series and that larger samples provide a more accurate representation.

While only a limited number of studies have explored reoperations after embolization, reporting less than or equal to 2 cases [3,12,14-16], our study stands out with 12 patients requiring reoperation, making it a significantly higher proportion than previously reported. Our findings suggest that while embolization is effective in the majority of cases, there may be underlying mechanical reasons for hemarthrosis that cannot be identified.

In our cohort, all embolizations were conducted under local anesthesia, but without the use of sedation or antibiotic prophylaxis, in contrast to other studies [13,14,17]. The most common method of pain management was achieved with oral administration of paracetamol and opioids. This finding aligns with previous research, suggesting that postprocedural pain, numbness, and discomfort are transient and can be alleviated with minimal use of analgesics [12]. These results imply that the prophylactic femoral nerve blockage employed in 10 patients may not be necessary in future procedures. Most patients were discharged on the first postoperative day, which is supported by the results of previous studies [3,12]. Based on this study, it is possible for patients to be discharged safely on the day of the procedure, as seen in 2 patients, as there were no readmissions due to pain and patients did not experience any major complications.

Complications can result from arterial puncture or excessive arterial embolization [5,12]. The meta-analysis by Sundaram et al. [8] reported a 19% incidence of complications; however, several other studies found no complications associated with embolization [18-20]. Our case series presented 5 complications, with 3 categorized as mild and 2 as moderate. Mild complications resolved spontaneously, which supports the general safety of the embolizations.

### Limitations

This study was limited by its retrospective design. We were unable to evaluate nonoperatively treated hemarthrosis because our data included only inpatient data on patients treated with specialized healthcare. As such, the retrospective design did not allow for collection of more extensive clinical data or a control group. Additionally, the embolization procedure cannot be completely standardized as the radiologist’s proficiency in performing embolization remains a significant confounding factor.

### Conclusions

AE is effective in the treatment of recurrent hemarthrosis but 21% had reoccurance. Oral analgesics are generally sufficient for managing post-embolization pain. 7% had complications.

## Notes

SS, TN, PK, and JK designed the study protocol. SS, TN, PK ,and JK participated in data acquisition. All authors contributed to interpretation of the results, manuscript writing, and editing.

Handling co-editor: Ivan Hvid

*Acta* thanks Rudi Georg Bitsch and Per Henrik Randsborg for help with peer review of this manuscript.
